# Surgical management of a benign multicystic peritoneal mesothelioma: A case report

**DOI:** 10.1016/j.ijscr.2023.108308

**Published:** 2023-05-09

**Authors:** Sebai Amine, Ouadi Yacine, Atri Souhaib, Jedidi Yasmine, Makni Amine, Montasser Kacem

**Affiliations:** Department of Surgery A La Rabta Hospital, Tunis, Tunisia; Faculty of Medicine of Tunis, Tunis El Manar University, Tunis, Tunisia

**Keywords:** Peritoneal mesothelioma, Cystic lymphangioma, Cystic mesothelioma, Benign mesothelioma, Mesothelioma, General surgery

## Abstract

**Introduction and importance:**

Benign multicystic peritoneal mesothelioma is rare, with around 200 cases reported in the literature.

We report the case of a patient operated on for the diagnosis of cystic lymphangioma but the pathology examination retained the diagnosis of benign cystic peritoneal mesothelioma.

**Case presentation:**

A 47-year-old patient, who consulted for abdominal distension evolving for a year. Examination revealed a 30-centimeter abdominal mass. The CT scan showed an intraperitoneal cystic mass measuring 24 × 13 × 32 cm. The diagnosis of cystic lymphangioma was suspected and we decided to surgically remove the mass. We performed a laparotomy. There was a large multi-cystic formation that seemed to develop at the expense of the parietal peritoneum and the greater omentum. A monobloc resection was performed. The postoperative was eventless. Pathology concluded to a benign cystic peritoneal mesothelioma.

**Discussion:**

The BMPM is a rare peritoneal neoplasm that develops mainly in women, during sexual activity. Its etiopathogenesis is unknown. It is often mesenteric or omental.

Generally, resection is considered the sole treatment for benign mesotheliomas. However, this surgery needs to be R0 or it will expose to a certain recurrence.

Some authors recommend a more aggressive approach associating cytoreductive surgery with heated intraperitoneal chemotherapy.

**Conclusion:**

Benign multicystic peritoneal mesothelioma is a rare pathology of the peritoneum which develops mainly in women during periods of reproductive activity. Despite its benignity, it presents a high risk of recurrence, up to 50 % of cases.

## Highlights

• Benign multicystic peritoneal mesothelioma is extremely rare.
• Pre-operative diagnosis is challenging.
• There is no consensus over its treatment.

## 1 Introduction and importance

Peritoneal tumors represent a heterogeneous group of different prognostic neoplasms, of which the most dreadful is the malignant peritoneal mesothelioma. Benign multicystic peritoneal mesothelioma (BMPM) is the rarest one, with around 200 cases reported in the literature [1]. It is a benign pathology with a difficult preoperative diagnosis. No clear consensus over its treatment is established due to its rarity.

Herein, we report the case of a patient operated on for a peritoneal cystic mass whose CT characteristics were consistent with those of a cystic lymphangioma. Pathological examination retained the diagnosis of benign cystic peritoneal mesothelioma. We report this case to enrich furthermore the literature with our experience and management of this disease.

This case report has been reported in line with the SCARE Criteria [2].

## 2 Case report

**Fig. 1:**
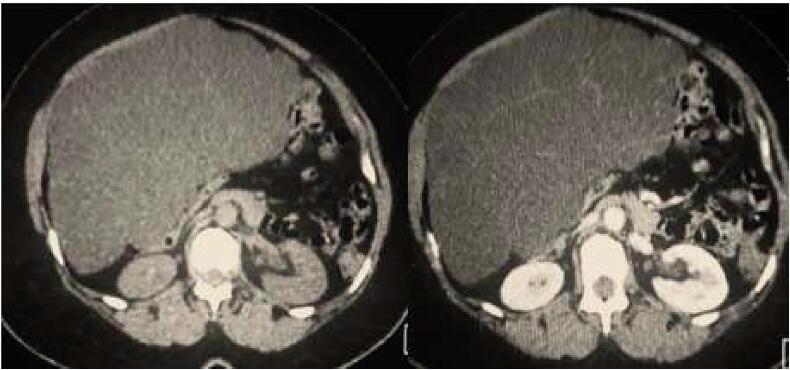
CT scan before and after contrast fluid injection: Multicystic peritoneal neoplasm displacing neighboring organs. Presence of thin walls slightly highlighted after contrast fluid injection.

It was a 47-years-old female patient, with no past medical history, who consulted for an abdominal distension evolving for one year. Examination found a 30-centimetre abdominal mass. It was painless, regular and fixed. CT scan showed a large intraperitoneal cystic mass of 24 ∗ 13 ∗ 32 cm. It had multiple thin walls slightly highlighted after contrast fluid injection (Fig. 1). It seemed to develop at the expense of the great omentum.

**Fig. 2:**
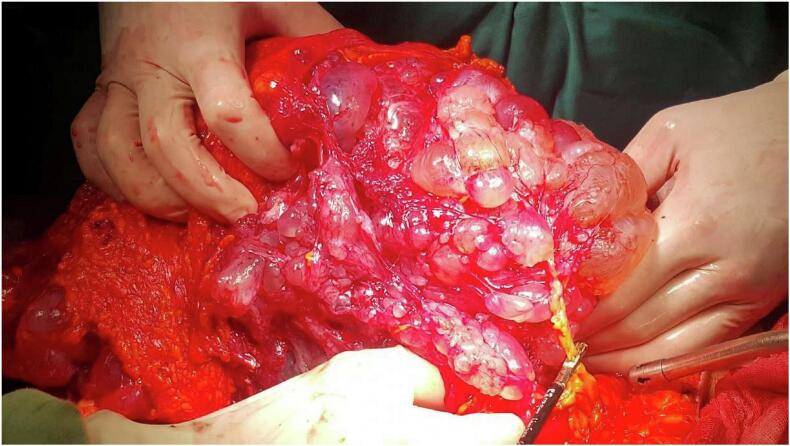
Epiploic multicystic neoplasm.

**Fig. 3:**
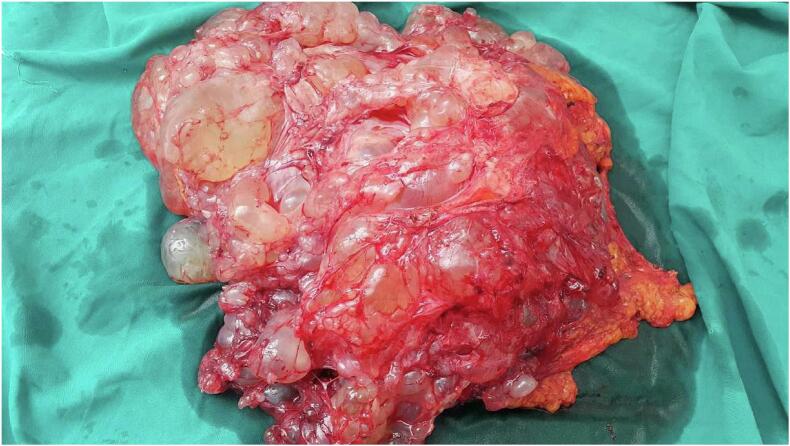
Multicystic mass with a crystal-clear liquid content.

The diagnosis of cystic lymphangioma was retained. Surgical resection was decided. We performed a mid-line laparotomy. There was a voluminous multi-cystic formation with clear liquid content. It seemed to develop at the expense of the parietal peritoneum and the great omentum. It didn't invade any abdominal structure (Fig. 2). These inoperative findings were more suggestive of a BMPM. Thus, an en-bloc resection of the tumor and great omentum was performed (Fig. 3). Postoperative courses were eventless. Pathological examination of the surgical specimen confirmed the diagnosis of benign cystic peritoneal mesothelioma.

In the past two years, the patient had regular monitoring at the outpatient clinic via clinical examination and CT scans every 6 months that showed no signs of recurrence so far.

## 3 Discussion

The benign cystic peritoneal mesothelioma (BMPM) is a rare peritoneal neoplasm. To the best of our knowledge, less than 200 cases are reported in the literature [3].

It develops mainly in women, during sexual activity, with very few reports of this disease in females over the age of 30 [4]. The fact that our patient was a 47-year-old woman is one factor that made our case report more valuable.

BMPM pathogenesis remains unclear. Nevertheless, it is commonly associated with pelvic inflammatory diseases, endometriosis, or a history of prior surgeries. This suggests that the chronic inflammation of the peritoneum is a crucial factor for the proliferation and metaplasia of mesothelial cells [5].

Symptoms are nonspecific as they are due to its mass effect: abdominal distension, abdominal and pelvic pain, digestive and urinary compression. This makes the diagnosis difficult as was the case for our patient.

On the CT-scan, BMPM presents usually as a hypodense, multicystic, and multilocular neoplasm, with thin septa displacing neighboring organs without invading them [6]. However, these findings are far from being specific to BMPM. Indeed, several cystic peritoneal lesions may be evoked when facing these aspects especially cystic lymphangiomas (CL) [7].

This makes the diagnosis even more challenging. For our patient, and despite a suggestive CT-scan, the diagnosis of BMPM wasn't evoked as these tumors are scarce and even more exceptional in a 47-year-old women with no past history of chronic peritoneal inflammation.

The rarity of BMPM makes consensus over its treatment impossible. The most recommended treatment is an R0 resection with HIPEC. This makes preoperative diagnosis mandatory in order to perform an optimal surgery. HIPEC is believed to extend disease-free survival. Indeed, R0 resection alone is associated with a recurrence rate of 50–60 %. However, no data is available to confirm this hypothesis. Due to this high recurrence rate, some authors recommend regular surveillance over surgery for asymptomatic patients [1], [3].

We believe that each case must be discussed in a multidisciplinary meeting in order to be able to make a la carte treatment for each patient.

## 4 Conclusion

Multicystic benign peritoneal mesothelioma is a rare pathology of the peritoneum that develops mainly in women during periods of genital activity. It has no radiologic specific signs, and is often confused with cystic lymphangioma. Its treatment is surgical, and the pathological examination of the operative specimen retains the diagnostic. Despite its benignity, it presents a high risk of recurrence, up to 50 % of cases.

## Consent

Written informed consent was obtained from the patient for publication of this case report and any accompanying images. A copy of the written consent is available for review by the Editor-in-Chief of this journal on request.

## Ethical approval

We confirm that ethical approval is exempt/waived at our institution from the ethical committee of La Rabta University Hospital of Tunis (Tunisia) for this case report.

## Funding

No sources of funding.

## Author contribution

Sebai Amine, Conceptualisation, Redaction, Data curation, Project administration

Ouadi Yacine, Conceptualisation, Redaction, Data curation, Project administration

Atri Souhaib Conceptualisation, Redaction

Jedidi Yasmine Photography Rendering, Data curation

Makni Amine Supervision, Validation, Visualisation

Montasser Kacem Supervision, Validation, Visualisation

## Guarantor

Yacine Ouadi.

## Research registration number

Not applicable.

## Provenance and per review

Not commissioned, externally pee-reviewed.

## Declaration of competing interest

All authors declare they have no conflict of interest.
